#  Prune Belly Syndrome with Situs Inversus Abdominus 

**DOI:** 10.21699/jns.v5i3.349

**Published:** 2016-07-03

**Authors:** Ganavi Ramagopal, Ganesh Narayana, Ashok Rathod

**Affiliations:** 1 Department of Pediatrics, JJ Group of Hospitals, Grant Medical College, Mumbai; 2 Department of Cardiology, JJ Group of Hospitals, Grant Medical College, Mumbai

**Dear Sir**

A full term male baby born to a 23-year-old primigravida did not develop spontaneous respiration after birth. His heart rate was less than 40/min and required bag and tube ventilation; and shifted to NICU for further management. Antenatal ultrasound at 36 weeks showed single live fetus with grossly distended bladder with dilated proximal urethra, bilateral hydroureters causing significant intra-abdominal mass effect with anhydramnios; findings were suggestive of a posterior urethral valve. On examination, baby had syndromic facies-low set ears, depressed nasal bridge, receding chin, hypertelorism, chest wall abnormality, excess of folds of skin on anterior abdominal wall (prune belly), (Fig. 1) epispadias, bilateral cryptorchidism, and Right congenital talipes equino varus (TEV) deformity. Baby was put on ventilator and ionotropic support started. X-ray abdomen and chest (Fig. 2) performed showed left sided pneumothorax and liver was on left side of abdomen, stomach bubble on right, floating ribs with scoliosis, and cardiac apex to the left side. Immediately needle aspiration was done and later a chest drain was put. The baby remained critical despite intensive management and succumbed to cardiopulmonary failure at 7thhr of life. Autopsy was deferred in view of no consent from parents.

**Figure:**
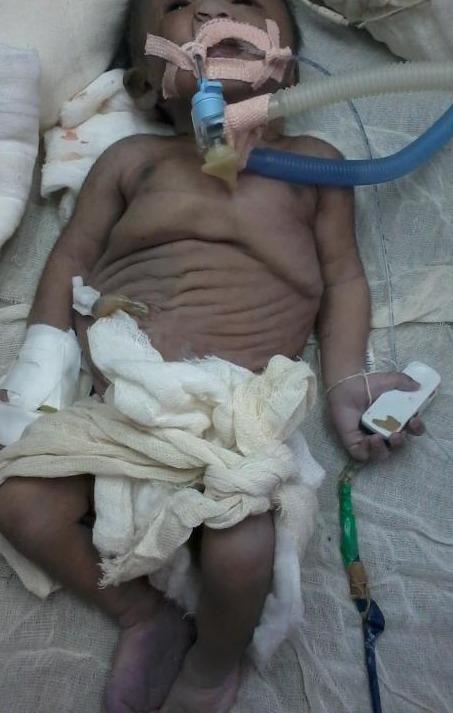
Figure 1: Baby with features of Prune belly syndrome.

**Figure:**
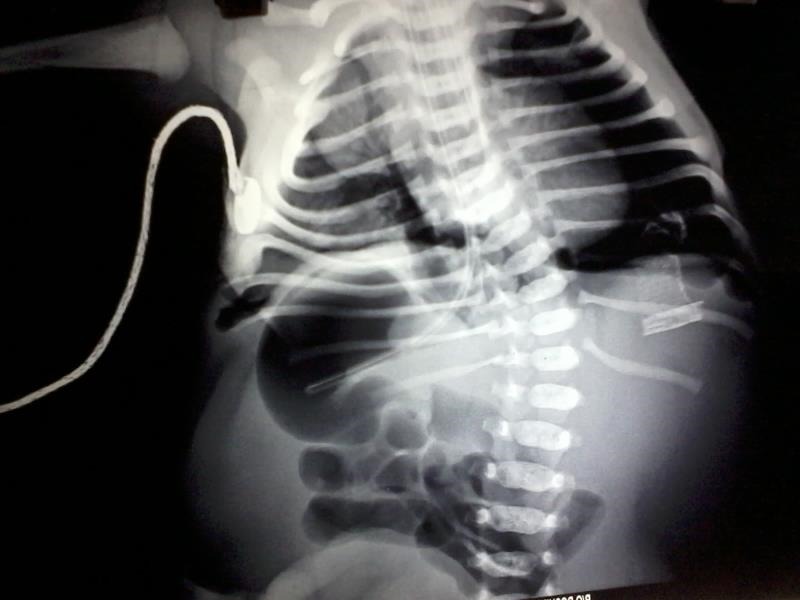
Figure 2: X-ray showing left pneumothorax, right lung hypoplasia, liver on left, stomach bubble on right, floating ribs and scoliosis.

Prune belly syndrome (PBS) is characterized by the triad of abdominal wall muscular defect, bilateral hydronephroureterosis, and cryptorchidism.[1] The mesodermal defect theory and the urethral obstruction malformation complex theory, tried to explain it pathogenesis and concurrence of its components but exact pathogenesis is not clearly known as yet.[2] Along with the classical triad of anomalies, PBS is also associated with a broad spectrum of defects including musculoskeletal, cardiovascular, pulmonary, genital, and gastrointestinal abnormalities.[2] In our case there were many abnormalities associated with PBS. The potter facies and lung hypoplasia were due to anhydramnios. The association of PBS with heterotaxy is extremely rare and only one case was reported so far by Xu et al. [3]

## Footnotes

**Source of Support:** Nil

**Conflict of Interest:** None
